# Revisiting atenolol as a low passive permeability marker

**DOI:** 10.1186/s12987-017-0078-x

**Published:** 2017-10-31

**Authors:** Xiaomei Chen, Tim Slättengren, Elizabeth C. M. de Lange, David E. Smith, Margareta Hammarlund-Udenaes

**Affiliations:** 1 Department of Pharmaceutical Biosciences, Translational PKPD Research Group, Uppsala University, Box 591, SE-75124 Uppsala, Sweden; 2 Department of Pharmaceutical Sciences, College of Pharmacy, University of Michigan, Ann Arbor, MI 48109 USA; 3 Department of Pharmacology, Leiden Academic Centre for Drug Research, Leiden, The Netherlands

**Keywords:** Atenolol, Blood–brain barrier, Microdialysis, Unbound equilibrium partition coefficient (K_p,uu,brain_), Unbound volume of distribution in brain (V_u,brain_), Passive permeability, Transporters, Pharmacokinetics, Lipophilicity

## Abstract

**Background:**

Atenolol, a hydrophilic beta blocker, has been used as a model drug for studying passive permeability of biological membranes such as the blood–brain barrier (BBB) and the intestinal epithelium. However, the extent of S-atenolol (the active enantiomer) distribution in brain has never been evaluated, at equilibrium, to confirm that no transporters are involved in its transport at the BBB.

**Methods:**

To assess whether S-atenolol, in fact, depicts the characteristics of a low passive permeable drug at the BBB, a microdialysis study was performed in rats to monitor the unbound concentrations of S-atenolol in brain extracellular fluid (ECF) and plasma during and after intravenous infusion. A pharmacokinetic model was developed, based on the microdialysis data, to estimate the permeability clearance of S-atenolol into and out of brain. In addition, the nonspecific binding of S-atenolol in brain homogenate was evaluated using equilibrium dialysis.

**Results:**

The steady-state ratio of unbound S-atenolol concentrations in brain ECF to that in plasma (i.e., K_p,uu,brain_) was 3.5% ± 0.4%, a value much less than unity. The unbound volume of distribution in brain (V_u, brain_) of S-atenolol was also calculated as 0.69 ± 0.10 mL/g brain, indicating that S-atenolol is evenly distributed within brain parenchyma. Lastly, equilibrium dialysis showed limited nonspecific binding of S-atenolol in brain homogenate with an unbound fraction (f_u,brain_) of 0.88 ± 0.07.

**Conclusions:**

It is concluded, based on K_p,uu,brain_ being much smaller than unity, that S-atenolol is actively effluxed at the BBB, indicating the need to re-consider S-atenolol as a model drug for passive permeability studies of BBB transport or intestinal absorption.

## Background

Atenolol is a selective beta receptor blocker for the treatment of hypertension with the enantiomer S-atenolol responsible for the main active pharmacological effect [1–3]. For a long time, atenolol has been considered as a typical representative of a hydrophilic small molecule with low passive permeability and low paracellular diffusion across intestinal membrane and blood–brain barrier (BBB). Thus, it has been used as a model drug in developing and evaluating in vitro or in situ models for intestinal absorption and CNS penetration [4–6].

Like the intestinal epithelium, the BBB is characterized by tight junctions formed between adjacent cerebral capillary endothelial cells. These restrict paracellular transport, a pathway important for ions and other small hydrophilic molecules, which thus have lower permeability across the BBB and enterocytes. On the other hand, tight junctions have a limited effect on the BBB and intestinal permeability for lipophilic molecules that mainly use the transcellular pathway [7].

There have been several in vivo methods developed to assess the rate of drug transport across the BBB, including intravenous injection to measure the BBB permeability surface area product, intra-arterial injection to measure the brain uptake index, as well as in situ brain perfusion to assess BBB permeability using well-controlled perfusate [8–10]. From the above methods, if the samples are collected at very early time points, drug transport from brain back to blood is considered to be low and thus negligible, in which case the rate of initial brain uptake can be specifically studied. However, this includes possible influences of efflux transporters on the rate of brain uptake. Instead of assessing transport rate across the BBB, microdialysis can be used to evaluate the rate as well as extent of drug transport by measuring unbound drug concentrations in the extracellular fluid (ECF) of brain tissues over a longer duration. By modeling the microdialysis data with the information of unbound drug volume of distribution in brain (V_u,brain_) the permeability in both directions, influx clearance into brain (CL_in_) and efflux clearance from brain (CL_out_), can also be estimated [11]. CL_in_ and CL_out_ values are determined by the contribution of both passive diffusion and active transport. Moreover, CL_out_ may be affected by metabolism and ECF bulk flow [12]. The ratio of CL_in_ over CL_out_ values, is equal to the unbound equilibrium partition coefficient, K_p,uu,brain_, which is defined as the ratio of unbound drug concentration in brain ECF to that in plasma at the steady state [13]. Even when steady state concentrations are not achieved, but with rate processes following first order kinetics (i.e. linear pharmacokinetics), K_p,uu,brain_ can be estimated using the ratio of area under curve of unbound drug concentration–time profiles (AUC_u_) in brain ECF to AUC_u_ in plasma. It should be noted that the K_p,uu,brain_ value reflects the *extent* of unbound drug concentration equilibration between brain and plasma, but not the rate with which a drug crosses the BBB [12]. Typically, BBB permeability is a measure of the *rate* of BBB transport of the drug. Compounds with lower lipophilicities tend to have lower BBB permeability, only if passive transport governs the exchange of drug molecules across the BBB.

For a drug with only passive transport across the BBB, it holds that CL_in_ = CL_out_ with respect to unbound drug, making K_p,uu,brain_ equal to unity. In other words, at steady state, the unbound drug concentration in brain ECF is equal to that in plasma. Drugs with a low BBB permeability just need more time to reach such equilibrium, but K_p,uu,brain_ is independent of BBB permeability [12].

If atenolol were a typical drug of low passive BBB permeability, it would have equal CL_in_ and CL_out_, leading to the following characteristics: (1) without any carrier-mediated transport or being metabolized in brain, its K_p,uu,brain_ value would be unity [12]; (2) as the net direction of mass transport for passive diffusion is only determined by unbound concentration gradient between the two sides of BBB, its unbound brain concentration would keep increasing when higher unbound concentrations are present in blood than in brain (i.e. C_u,blood_ > C_u,brain_) and C_u,brain_ would start decreasing when C_u,blood_ < C_u, brain_. However, a previous microdialysis study of atenolol in rats showed a ratio of AUC_u_ in brain ECF to AUC in plasma of only 3.8 ± 0.6% after an intravenous 10 mg bolus dose. In addition, the peak of the C_u,brain_ was at around 10 min, when the plasma concentration was much higher than C_u,brain_. Moreover, both unbound brain and plasma concentration–time profiles had the same half-lives [14]. This is not consistent with the expected profile described above for compounds with only passive permeability. Instead, the reported C_u, brain_-time profile of atenolol resembles that of compounds with active efflux, based on the simulations performed by Hammarlund-Udenaes et al. [15].

If indeed atenolol has a very low K_p,uu,brain_ due to it being a substrate of an efflux transporter, it has important implications on the role of atenolol as a model drug for low passive permeability (i.e. low paracellular diffusion without any carrier-mediated transport), and thus the conclusions from the related research of biological membrane barriers may need reevaluation. Therefore, the aim of this study was to investigate in-depth the in vivo net flux of S-atenolol BBB transport. To that end, a detailed microdialysis study was carried out to evaluate the K_p,uu,brain_ of S-atenolol, and investigate its intra-brain distribution by assessing the V_u,brain_ and the unbound drug fraction in brain homogenate (f_u,brain_). Modeling and simulation were used to describe the properties of atenolol from a rate and extent perspective.

## Methods

### Chemicals

S-(−)-atenolol and atenolol-D7 were purchased from Sigma-Aldrich (St. Louis, MO, USA). Isoflurane was obtained from Baxter Medical AB (Kista, Sweden). Ringer’s solution was prepared to perfuse microdialysis probes and comprised 145 mM NaCl, 0.6 mM KCl, 1.0 mM MgCl_2_, 1.2 mM CaCl_2_, and 0.2 mM ascorbic acid in 2 mM phosphate buffer (pH 7.4). Normal saline was obtained from Braun Medical AB (Stockholm, Sweden), and water was purified using a Milli-Q system (Millipore, Bedford, MA, USA). Ammonium acetate and acetonitrile were purchased from Merck (Darmstadt, Germany). All other chemicals were of analytical grade.

### Animals

Male Sprague–Dawley rats (250–310 g) were obtained from Taconic (Lille Skensved, Denmark). The animals were acclimated for 1 week before the experiment and housed in groups with 12-hour day-night cycles at 22 °C. The microdialysis study was approved by the Animal Ethics Committee of Uppsala University, Sweden (C328/10).

### Microdialysis study

For the microdialysis study, vessel catheters and microdialysis probes were implanted in rats as previously described [13, 16]. Briefly, the rats were anesthetized using 2.5% isoflurane and their body temperature were maintained at 37 °C using CMA/150 temperature controller (CMA, Stockholm, Sweden) throughout the surgery. Firstly, a catheter made from PE-50 fused with silicon tubing was implanted into the femoral vein for S-atenolol infusion, followed by the insertion of a PE-50 catheter fused with PE-10 into the femoral artery for blood sampling. Secondly, an incision was made to insert a CMA/20 microdialysis probe (CMA, Stockholm, Sweden) with 10 mm flexible polyarylethersulphone (PAES) membrane into the right jugular vein for sampling unbound S-atenolol in plasma. Then, the head of the rat was fixed on a stereotaxic frame and a guide cannula was implanted into striatum with the coordinates 0.8 mm anterior, 2.7 mm lateral to the bregma, and 3.8 mm ventral to the surface of the skull. Dental cement was used to fix the guide cannula onto the skull with an anchor screw. The tubing of the vessel catheters and microdialysis probe were tunneled subcutaneously and fixed at the back of the neck. At the end of the surgery, the dummy inside the guide cannula was replaced by a CMA/12 microdialysis probe (CMA, Stockholm, Sweden) with a 3 mm PAES membrane (20 kDa cutoff) for sampling S-atenolol in brain ECF. The rats were allowed to recover for 1 day before the microdialysis study and to move freely in a CMA 120 system with free access to food and water.

**Fig. 1:**
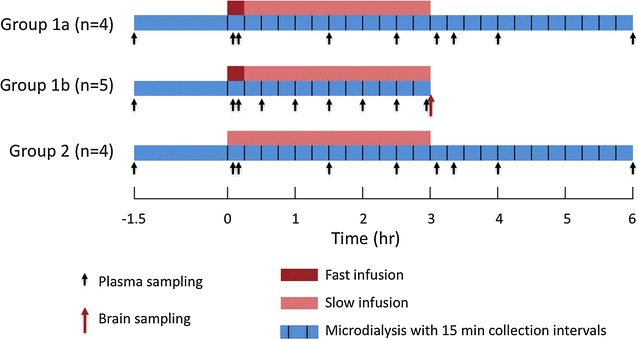
Design of the microdialysis study of S-atenolol showing the time aspects of i.v. infusion (red and pink bars), microdialysis sampling (blue bars), plasma sampling (black arrows), and brain tissue sampling (red arrow)

As shown in Fig. 1, the rats were divided into two groups with different dosing regimens. The infusion solution had a drug concentration of 5 mg/mL. Group 1 (n = 9) received S-atenolol starting with a fast infusion at 0.4 mg/min/kg for 15 min followed by a slow infusion of 0.182 mg/min/kg for 165 min using a Harvard 22 pump (Harvard Apparatus Inc., Holliston, MA, USA) in order to rapidly achieve steady state concentrations in plasma. Samples were collected for another 3 h after the end of drug infusion in four rats (Group 1a). The rats in Group 1b (n = 5) were decapitated at the end of the infusion to harvest the brains in order to measure the total S-atenolol amount in brain tissue. In Group 2 (n = 4), S-atenolol was given as a single constant infusion for 3 h at a rate of 0.167 mg/min/kg, and continuing sampling for 3 h thereafter. In all rats, the microdialysis perfusion was started at the beginning of the stabilization period, 90 min before S-atenolol dosing. Deuterated atenolol, atenolol-D7, was used to measure the relative recovery across the microdialysis probes throughout the study, using retrodialysis by the atenolol-D7 as a calibrator [17, 18]. Atenolol-D7 was added to the Ringer’s solution at 50 ng/mL for brain probe and at 200 ng/mL for plasma probe, which were perfused through the microdialysis probes using a CMA 400 pump (CMA, Solna, Sweden) at a flow rate of 1 µL/min. The dialysates were collected every 15 min by a fraction collector (CMA 142, Solna, Sweden) until the end of experiment. For the animals with their drug elimination phase monitored, 100 µL of blood was drawn from the femoral artery pre-dose and at 5, 10, 90, 150, 185, 200, 240, and 360 min after the start of S-atenolol infusion. For the rats decapitated at the end of drug infusion, the blood was collected pre-dose and at 5, 10, 30, 60, 90, 120, 150, and 175 min. All blood samples were centrifuged at 7200*g* for 5 min to obtain plasma, which together with brain and microdialysis samples were frozen at − 20 °C until analysis.

## Equilibrium dialysis study

The f_u,brain_ at three drug concentrations was measured in vitro using equilibrium dialysis of brain homogenate. Briefly, Sprague–Dawley rats were decapitated under isoflurane anesthesia and the brains were collected and homogenized in four volumes of 180 mM phosphate buffer. After being spiked with 132.5, 265, and 1325 ng/mL S-atenolol (corresponding to 0.5, 1, and 5 µM), 150 µL of the blank homogenate was dialyzed against PBS pH 7.4 for 6 h using a Pierce Rapid Equilibrium Dialysis Device (RED) (Thermo Scientific, Rockford, IL, USA) (n = 5 at each concentration) with a shaking speed of 200 rpm at 37 °C (MaxQ4450, Thermo Fisher Scientific, Nino Lab, Sweden). Samples were collected from both buffer and homogenate sides at the end of the incubation period of 6 h. The stability of S-atenolol in brain homogenate was evaluated by incubating homogenate containing the drug at the three concentrations and collecting samples before and after the incubation. In order to obtain the same matrix for all samples in the chemical assay, the same volume of buffer was added to brain homogenate samples and vice versa. All samples were stored at − 20 °C until assay. The unbound fraction of S-atenolol in diluted brain homogenate (f_u,hD_) was calculated from the buffer/homogenate concentration ratio as:

1 $$ f_{u,hD} = \frac{{C_{buffer} }}{{C_{homogenate} }} $$

The unbound fraction of S-atenolol in brain was calculated according to Eq. 2 after correction for the dilution factor D associated with the preparation of brain homogenate (D = 5 in this study):

2 $$ f_{u,brain} = \frac{1}{{1 + D\left( {\frac{1}{{f_{u,hD} }} - 1} \right)}} $$

### Chemical analysis

Liquid chromatography coupled with tandem mass spectrometry (LC–MS/MS) was used to determine the concentrations of S-atenolol and atenolol-D7 in the microdialysis samples. Five microliters of the brain microdialysis samples were directly injected into the system. The plasma dialysate samples (15 µL) having high drug concentrations were diluted by adding 150 µL Ringer’s solution before analysis. After thawing to room temperature, the plasma samples were precipitated at a ratio of 1:3 with acetonitrile containing 500 ng/mL atenolol-D7 as internal standard. Following vortex mixing and centrifugation for 3 min at 7200*g*, 25 µL of the supernatant was further diluted by mixing it with 1 mL of 5 mM ammonium acetate solution and then injecting 10 µL of the mixture into the LC–MS/MS. The brain samples were homogenized with a tissue-saline ratio of 1:4 (w/v), prepared as described above. Then 150 µL of the homogenate was mixed with 150 µL of 50 ng/mL atenolol-D7 aqueous solution, and further precipitated with 150 µL acetonitrile. After 3 min centrifugation at 7200*g*, the supernatant was diluted tenfold with 5 mM ammonium acetate, injecting 50 µL. The homogenate samples from equilibrium dialysis were prepared with the same procedures as above. Standard curves were generated for all types of biological matrix (i.e., 0.5–500 ng/mL for dialysate; 50–10,000 ng/mL for plasma; 25–1000 ng/g brain for brain tissues from microdialysis study; 6.25–875 ng/mL for brain homogenate samples from equilibrium dialysis study) and quality control samples at low, medium and high concentrations were analyzed along with the samples for measurement validation. The coefficients of determination (r^2^) were ≥ 0.994 for all standard curves.

The LC–MS/MS system consisted of two Shimadzu LC-10ADvp pumps (Shimadzu, Kyoto, Japan), a SIL-HTc autosampler (Shimadzu, Kyoto, Japan), and a Quattro Ultima mass spectrometer (Waters, Milford, MA, USA). A HyPurity C18 column (50 × 4.6 mm, 3 µm particle size), equipped with a HyPurity C18 guard column (10 × 4.0 mm, 3 µm particle size, Thermo Scientific Hypersil-Keystone, PA, USA), was used for chromatographic separation with a gradient elution involving mobile phase A (5 mM ammonium acetate in water) and mobile phase B (90:10 v/v acetonitrile:water). The flow rate was set to 0.8 mL/min, which was split to 0.3 mL/min before entering the mass spectrometer, where positive electrospray ionization (ESI +) was applied. The transition mode was *m/z* 266.9 → 145 for S-atenolol and *m/z* 273.8 → 145 for atenolol-D7. All chromatographs were acquired and analyzed using Masslynx 4.0 (Waters, Milford, MA, USA).

### Calculations and pharmacokinetic data analysis

The relative recovery of S-atenolol for each microdialysis probe was evaluated using retrodialysis with atenolol-D7 as a calibrator according to

3 $$ Recovery = \frac{{C_{in,ATD7} - C_{out,ATD7} }}{{C_{in,ATD7} }} $$

where C_in,ATD7_ and C_out,ATD7_ are the concentrations of atenolol-D7 in perfusate and dialysate, respectively [18]. The relative recovery simultaneously determined by the retrodialysis of atenolol-D7 was 6.94 ± 0.67% for the microdialysis probes in brain and 50.1 ± 1.9% for the probes in blood without any time-dependence. The unbound concentration of S-atenolol in brain ECF and plasma was calculated by dividing the measured S-atenolol concentration in dialysate by the relative recovery.

The K_p,uu,brain_ was calculated to characterize the extent of S-atenolol equilibration across the BBB as:

4 $$ K_{p,uu,brain} = \frac{{C_{u,ss,brainECF} }}{{C_{u,ss,plasma} }} $$

where C_u,ss,brainECF_ and C_u,ss,plasma_ are the unbound drug concentrations in brain ECF and plasma at the steady state, respectively.

The half-lives in brain ECF and plasma, t_1/2,brainECF_ and t_1/2,plasma_, were calculated based on the corresponding middle time points of microdialysis collection intervals of the elimination phase:

5 $$ t_{1/2} = \frac{0.693}{{\lambda_{z} }} $$

where λ_z_ is the terminal rate constant obtained from the last seven observations. The half-lives of unbound S-atenolol in brain ECF and plasma were compared using paired t test.

A pharmacokinetic model was developed using nonlinear mixed effect modeling (NONMEM, version 7.3.0, ICON Development Solutions, Ellicott City, MD, US) to describe the rate of S-atenolol transport across the BBB via CL_in_ and CL_out_. The method of first-order conditional estimation with interaction (FOCEI) was used throughout the modeling procedure. The inter-individual variability was investigated for all pharmacokinetic parameters during the model development using an exponential model:

6 $$ P_{i} = P_{pop} e^{{\eta_{i} }} $$

where P_i_ is the value of the parameter for the i-th individual, while P_pop_ is the typical value of the parameter in the population. The inter-individual variability was described by η, which was assumed to follow a normal distribution with a mean at 0 and standard deviation ω. In addition, different error models (proportional, additive, and slope-intercept error models) were explored to evaluate the residual variability, i.e. the difference between predicted and observed concentrations, for each type of observations.

The model selection was based on the objective function value (OFV), model parameter precision and graphical analysis. The likelihood ratio test was used to compare between nested models. Specifically, the difference in OFV between two nested models asymptotically follows χ2 distribution, and a drop in OFV of ≥ 3.84 indicates the superiority of the model for one-parameter difference with p ≤ 0.05. The parameter precision was described by relative standard error, RSE %, which was calculated as the standard error (S.E.) divided by the parameter estimate. The graphical analyses were performed using PsN (version 4.4.0, Uppsala University, Uppsala, Sweden) and Xpose 4 (version 4.5.3, Uppsala University, Uppsala, Sweden) together with R (version 3.3.1, R Foundation for Statistical Computing, Vienna, Austria).

**Fig. 2:**
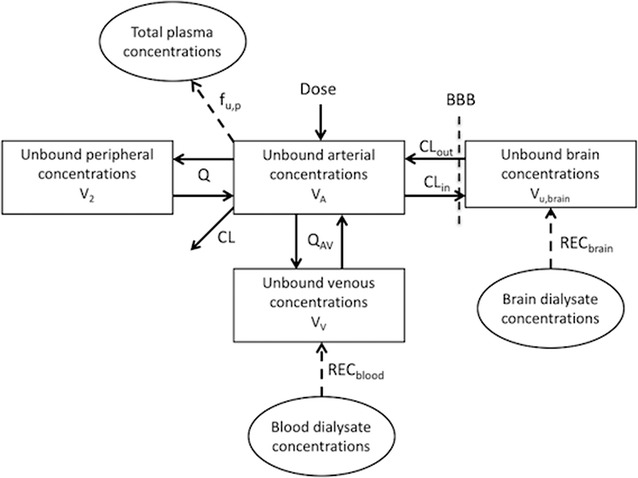
Schematic illustration describing pharmacokinetics and brain distribution of S-atenolol, and transformation of the microdialysis data by evaluating the probe recoveries. Solid arrows show mass transport between compartments (squares). Dashed arrows represent the transformations and corrections from observed dialysate data (ovals) to the unbound drug concentration in brain and plasma. Relative recoveries (REC), systemic total clearance (CL), clearance between arterial and peripheral compartments (Q), clearance between arterial and venous compartments (Q_AV_), volume of distribution of the arterial compartment (V_A_), volume of distribution of the venous compartment (Vv), volume of distribution of the peripheral compartment (V_2_), unbound fraction in plasma (f_u,p_), influx clearance into brain (CL_in_), efflux clearance out of brain (CL_out_), and unbound volume of distribution in brain (V_u,brain_)

The previously developed integrated plasma-brain pharmacokinetic model for oxymorphone, oxycodone, and DAMGO was used in this study, with modification based on the data from the microdialysis study of S-atenolol [13, 19, 20]. All observed data of S-atenolol were included in the model comprising total plasma concentration in arterial sampling, unbound concentration in venous plasma from microdialysis sampling in jugular vein, and unbound concentration in brain ECF from microdialysis sampling in right striatum (Fig. 2). The model also took into account the relative recovery by including the concentrations of the calibrator atenolol-D7 in dialysate from both probes.

The model development started by building a plasma PK model, followed by adding the other compartments in steps. The parameters in the final model were estimated simultaneously based on all data. In the model, the central compartment was divided into two compartments, an arterial compartment for plasma concentration and a venous compartment for microdialysis sampling. The two compartments were assumed to have equal unbound volume of distribution, that is, VA = VV. The transport of S-atenolol across the BBB was parameterized by CL_in_ and K_p,uu,brain_, which were assessed according to:

7 $$ CL_{in} = k_{in} \cdot VA $$

8 $$ K_{p,uu,brain} = \frac{{CL_{in} }}{{CL_{out} }} $$

9 $$ CL_{out} = k_{out} \cdot V_{u,brain} $$

where k_in_ and k_out_ denote the rate constants between the arterial compartment and the brain compartment. V_u, brain_ (mL/g brain) reflects the drug distribution within brain parenchyma since it describes the relationship between the total drug amount in brain and the unbound drug concentration in brain ECF:

10 $$ V_{u,brain} = \frac{{A_{brain} - C_{p} \times V_{bl} \times R_{bl - p} }}{{C_{u,ECF} }} $$

where A_brain_ is the measured drug amount in brain and C_p_ is the plasma concentration at the end of infusion. The volume of vascular space in rat brain (V_bl_) is 0.014 mL/g brain [21], and the blood-to-plasma concentration ratio of atenolol (R_bl-p_) is reported as 1.07 [22].

In order to illustrate the difference between efflux-transported drug and a drug with only passive diffusion across the BBB, simulations were performed for the cases: (1) CL_in_ = CL_out_ and (2) CL_in_ < CL_out_ with a constant i.v. infusion of 0.167 mg/min/kg (assuming a 280-g rat). The PK parameters were set as the typical values obtained from S-atenolol modeling.

All data are expressed as mean ± SEM in this report and GraphPad Prism v5.04 (GraphPad Software Inc., San Diego, CA) was used for statistical analysis and plots.

## Results

### Microdialysis study

**Fig. 3:**
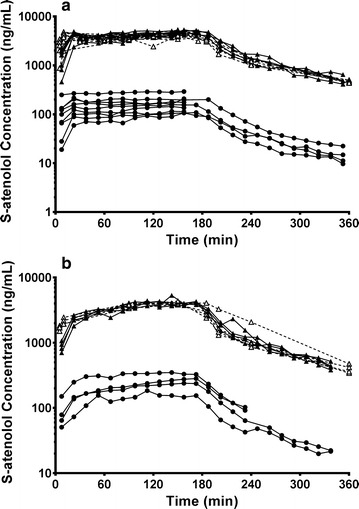
Individual concentration–time profiles of unbound S-atenolol in plasma (solid triangles and line) and brain (solid circles and lines) as well as total S-atenolol in plasma (open triangles and dashed lines) for (**a**) Group 1a and b (n = 9) with 15-min fast i.v. infusion followed by 165-min slow i.v. infusion, and (**b**) Group 2 (n = 4) with constant slow i.v. infusion for 180 min. For two rats, the C_u,brain_ data after 240 min are missing due to an LC–MS/MS malfunction during the analysis

In Group 1, the unbound S-atenolol concentration in plasma increased quickly during the 15-min fast infusion and was maintained at steady state (C_u,ss,plasma_) during the following 165 min slow infusion (Fig. 3a). The concentrations in plasma were comparable to the unbound S-atenolol concentration in plasma, indicating little to no binding of drug in plasma (f_u,p_ approaches 1). The steady state unbound concentration of S-atenolol in brain ECF was also quickly achieved and the concentration–time profile during elimination phase exhibited a similar shape to that in plasma. However, the brain ECF concentrations were much lower than in plasma throughout the whole experiment. The unbound S-atenolol steady-state concentration in plasma calculated from 90 to 180 min was 4429 ± 94 ng/mL, nearly 30-fold higher than in brain ECF (158 ± 20 ng/mL). The concentration–time profile of atenolol in Group 2 for the 3 h constant i.v. infusion followed a similar pattern (Fig. 3b). The unbound S-atenolol level gradually increased during the infusion in plasma and brain ECF to 4127 ± 103 ng/mL and 256 ± 41 ng/mL, respectively, at the last time point before the infusion ended.

**Fig. 4:**
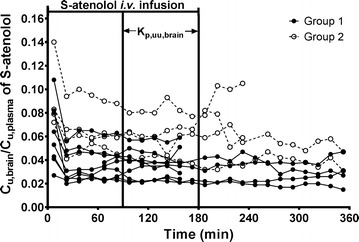
The ratio of unbound S-atenolol in rat brain ECF to that in plasma (C_u,brain_/C_u,plasma_) versus time for Group 1 (solid circles and lines) with 15-min fast i.v. infusion followed by 165-min slow i.v. infusion (n = 9) and for Group 2 (open circles and dashed lines) with 180-min constant i.v. infusion. The unbound partition coefficient (K_p,uu,brain_) was calculated during steady state (between 90 and 180 min) for Group 1

There was a rapid exchange and equilibration of S-atenolol across the BBB in spite of its low passive permeability. For both groups during the elimination phase, brain ECF concentrations decreased at the same rate as in plasma, which was confirmed by similar terminal half-lives in brain ECF and plasma (82 ± 7 min vs 85 ± 10 min, p = 0.325, paired t-test). In addition, the unbound brain to plasma ratio with time was stable both during the infusion period and during the elimination phase (Fig. 4). The K_p,uu,brain_ of S-atenolol was 3.55% ± 0.40% during 90–180 min.

The V_u,brain_ of S-atenolol was 0.686 ± 0.104 mL/g brain calculated from Eq. 10, which was not significantly different from the brain total water volume (0.8 mL/g brain) (p = 0.137). This suggested an even distribution of atenolol in brain with nonsignificant binding to brain parenchymal tissue and similar drug concentration in brain ECF and intracellular fluid (ICF) [12].

### Equilibrium dialysis study

From the equilibrium dialysis of brain homogenates, it was found that the f_u,brain_ of S-atenolol was 0.74 ± 0.04, 0.80 ± 0.04, and 1.09 ± 0.15 at the S-atenolol incubation concentrations of 0.5, 1.0, and 5.0 µM, respectively. There was no significant difference among the three S-atenolol levels with p = 0.0833 from one-way ANOVA analysis, suggesting that the nonspecific binding of S-atenolol in brain homogenate was independent of the incubation concentration. The average f_u,brain_ from all the three concentration groups was 0.875 ± 0.067, comparable with a previously reported value of 0.90 ± 0.052 [23], indicating very limited binding in brain homogenate, in line with the V_u,brain_ estimates presented above. S-atenolol was very stable in brain homogenate with zero degradation (100 ± 1% recovery) during the 6 h incubation at 37 °C.

### Pharmacokinetic modeling

**Fig. 5:**
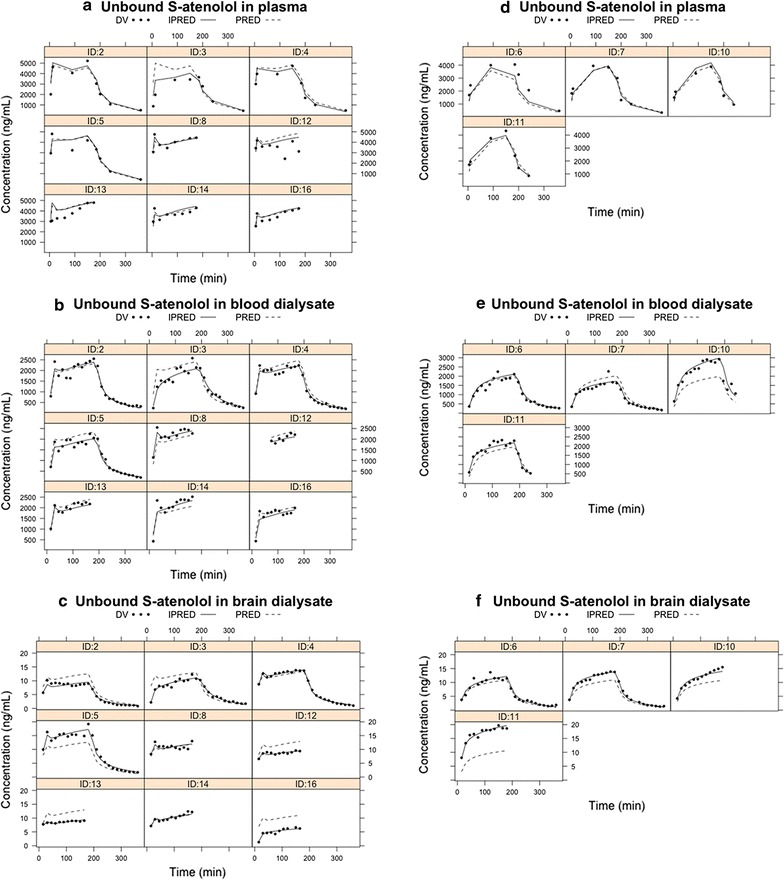
Individual plots of the concentrations of S-atenolol in plasma (**a**, **d**), blood dialysate (**b**, **e**), and brain dialysate (**c**, **f**) for Group 1 with 15-min fast i.v. infusion followed by 165-min slow i.v. infusion (**a**–**c**) and Group 2 with constant i.v. infusion for 180 min (**d**–**f**). Plots show observations (DV, solid circles), individual predictions (IPRED, solid lines), and population predictions (PRED, dash lines) from the model for each animal

**Fig. 6:**
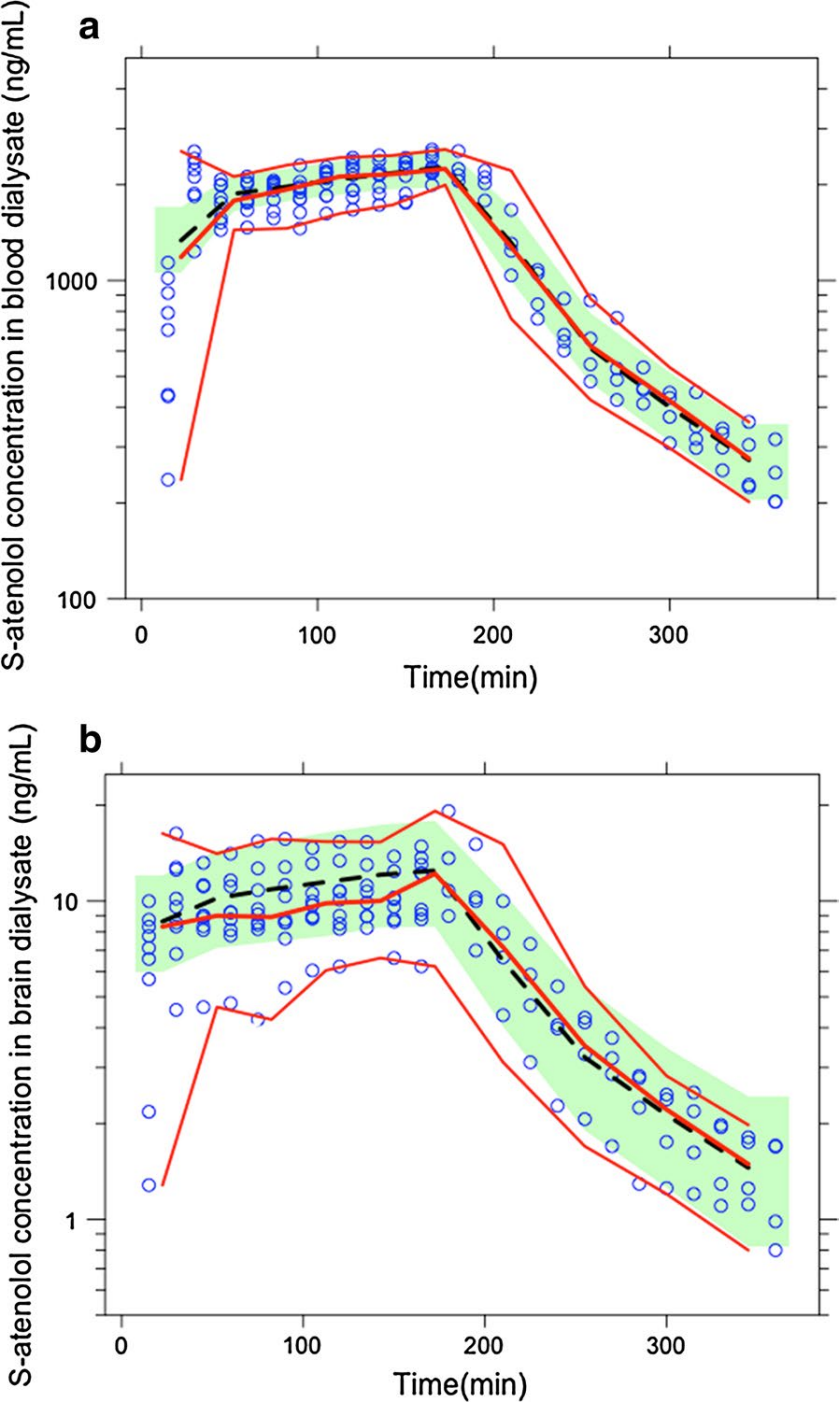
Visual predictive check for the final pharmacokinetic model based on 200 simulations for S-atenolol concentrations in blood dialysate (**a**) and in brain dialysate (**b**). The pharmacokinetic model involves the transformation of microdialysis data by evaluating probe recoveries, and thus the observed data for the model are the uncorrected drug concentrations in dialysate. Blue circles: observed data; red lines: median and 5th and 95th percentiles for observed data; black dashed line: median line of simulated data; green area: 95% confidence interval for the median simulated data

To be able to calculate the BBB clearance values, and to better understand the kinetics of S-atenolol transport at the BBB, a pharmacokinetic model including a brain compartment was developed based on the microdialysis data. The individual plots in Fig. 5 show observations, individual predictions and population predictions of S-atenolol in plasma, blood dialysate, and brain dialysate. A noticeable discrepancy between population and individual profiles was observed for some individuals (e.g. ID11 in brain dialysate), which may explain the large inter-individual variation for some parameters (Table 1). Nevertheless, the model is appropriate for describing S-atenolol distribution in plasma and brain, given the close median lines of real data and model-based simulation data in the visual predictive check based on 200 simulations (Fig. 6). The typical values of relative recoveries estimated from the model that included atenolol-D7 concentrations in dialysates are comparable to the values calculated directly from Eq. 1, and the model-estimated K_p,uu,brain_ of 4.00% is also comparable to the value of 3.55% from Eq. 4. CL_in_ is estimated as 17.0 µL/min/g brain, and the resultant CL_out_ is 425 µL/min/g brain based on the definition of K_p,uu,brain_, as the ratio of CL_in_ to CL_out_.

**Table 1:** Parameter estimates of the S-atenolol pharmacokinetic model in rats

Parameter	Unit	Estimate	RSE (%)	IIV (%)	RSE IIV (%)
REC_blood_	%	49.9	3.5	12.2	24.8
REC_brain_	%	6.73	9.5	27.9	17.2
CL	mL/min	10.2	2.4	7.5	16.9
V_1_	mL	215	10.8	30.3	28.4
Q	mL/min	5.56	8.9		
V_2_	mL	402	4.8		
f_u,p_		1.0	Fixed		
Q_AV_	mL/min	15.4	9.2		
CL_in_	µL/min/gbrain	17.0	48.8	134.2	27.5
K_p,uu,brain_		0.040	11.3	35.5	18.0
V_u,brain_	mL/g brain	0.686	Fixed		
σ_proportional,RECbrain_		0.028	9.4		
σ_additive,RECblood_	ng/mL	7.83	5.1		
σ_proportional,plasma_		0.184	20.3		
σ_proportional,blood_		0.112	8.8		
σ_proportional,brain_		0.0741	12.3		
σ_additive,brain_	ng/mL	0.22	20.2		

*RSE* relative standard error; *IIV* Inter-individual variation expressed as coefficient of variation; *REC* relative recoveries; *CL* systemic total clearance; *V*_*1*_ volume of distribution of total arterial and venous compartments; *Q* clearance between arterial and peripheral compartments; *V*_*2*_ volume of distribution of the peripheral compartment; *f*_*u,p*_ unbound fraction in plasma; *Q*_*AV*_ clearance between arterial and venous compartments; *Cl*_*in*_ influx clearance into brain; *K*_*p,uu,brain*_ unbound partition coefficient in brain; *V*_*u,brain*_ unbound volume of distribution in brain; *σ* variances of the proportional or additive residual errors

**Fig. 7:**
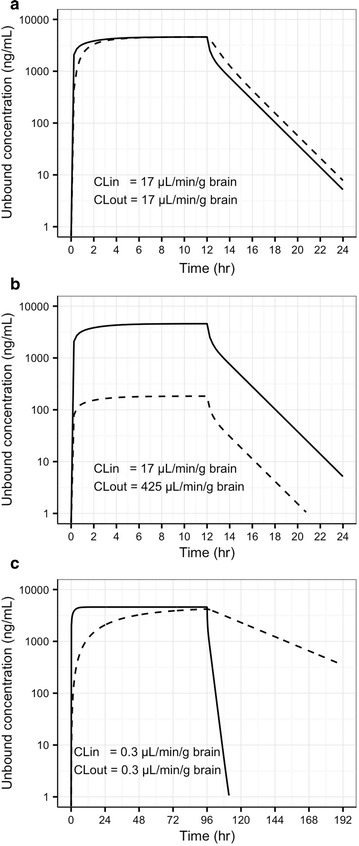
Simulation of unbound S-atenolol concentrations in arterial plasma (solid line) and in brain ECF (dashed line) for the scenarios of **a** CL_in_ = CL_out_ = 17 µL/min/g brain, **b** CL_in_ < CL_out_, and **c** CL_in_ = CL_out_ = 0.3 µL/min/g brain (i.e. permeability surface area product of sucrose) with an i.v. infusion of 0.167 mg/min/kg

To illustrate the unbound concentration profile for a compound with only passive diffusion across the BBB, simulation was performed by assuming CL_out_ = CL_in_ (17 µL/min/g brain) with a 12-h i.v. infusion (Fig. 7a). In this case, the unbound drug concentration is equal in plasma and brain at the steady state. Also, brain concentration decreased at a slower rate than the plasma concentration immediately after the infusion termination. On the other hand, a simulation was performed using the CL_in_ and CL_out_ values (17 and 425 µL/min/g brain, respectively) as estimated from the model of S-atenolol for the case of CL_in_ < CL_out_ and as a result there is a considerable difference between C_u,brain_ and C_u,plasma_ (Fig. 7b) during and after the drug infusion. The simulation was also performed based on the permeability surface area product of sucrose across the BBB (0.3 µL/min/g brain) [24]. Sucrose is a well-known marker for low intrinsic permeability without any active transport (Fig. 7c). Due to the lack of pharmacokinetic information of sucrose as well as our focus on the impact of BBB transport (CL_in_ and CL_out_), the model structure and the other parameter estimates used for sucrose simulation were the same as those for S-atenolol. The bulk flow was not considered in the simulation as no study has been found to quantify its impact on drug elimination from brain ECF. Compared to the scenario of CL_in_ = CL_out_ = 17 µL/min/g brain (Fig. 7a), the unbound brain concentration of sucrose in Fig. 7c takes much longer time to achieve 90% of steady state (3.7 days vs. 4 h) and has much longer half life during the elimination phase.

## Discussion

Beta blockers exhibit highly variable lipophilicity and accordingly diverse pharmacokinetic properties [25], catching the attention of scientists who study drug permeability across biological barriers. Therefore, the hydrophilic and lipophilic extremes in the beta blocker class, respectively, atenolol (logP of 0.23) and propranolol (logP of 3.65) have been used to study the relationship between lipophilicity and permeability in intestinal absorption and BBB penetration [25–27]. In addition, substantial efforts have been made to develop a variety of models to study and predict drug permeability, e.g. the in vitro Caco-2 cell model for intestinal absorption and in vitro brain capillary endothelial cell models for BBB transport. To evaluate and characterize these models, atenolol and propranolol are commonly used as model drugs for studying hydrophilic and lipophilic passive diffusion, respectively [5, 28, 29]. In addition to passive diffusion, carrier-mediated transport also plays a critical role in drug transport across biological barriers [30, 31]. Due to its importance, the function of transporters is usually evaluated by studying drug permeability across biological membranes in various in vivo, in situ, and in vitro models. In this context, atenolol is still used as a model drug for low passive/paracellular diffusion in permeability-related studies without further systematic assessment of the possibility of it being a transporter substrate.

The current study monitored, for the first time, the unbound concentration of S-atenolol in brain ECF during steady state and estimated its K_p,uu,brain_ to assess whether it is likely that any transporter is participating in the atenolol transport across the BBB. If atenolol is a hydrophilic drug without any involvement of transporters, it should have the profile of passive diffusion as in Fig. 7a with equal unbound concentration in plasma and brain at steady state. However, the present microdialysis study showed a profile with the S-atenolol K_p,uu,brain_ much lower than unity (3.55 ± 0.40%), measured at steady state. The C_u,brain_/C_u,plasma_ was stable during both the steady state and the elimination phases (Fig. 4), and corresponds to the AUC ratio of brain ECF to plasma from a previous microdialysis study after an intravenous bolus dose (3.8 ± 0.6%) [14]. The lower-than-unity K_p,uu,brain_ suggests that efflux transporters are involved in the atenolol transport at the BBB, leading to a higher CL_out_ than CL_in_. From the modeling approach, the CL_in_ value of atenolol in rats was 17.0 µL/min/g brain (Table 1), much lower than the CL_out_ of 425 µL/min/g brain (calculated according to Eq.  8). It should be noted that brain ECF bulk flow and metabolism may also contribute to discrepancies between CL_in_ and CL_out_ [12]. However, atenolol was found to be very stable in brain homogenate, thereby concluding that metabolism is not important. The relatively low bulk flow reported in rats of 0.1–0.3 µL/min/g brain [32, 33] is also of minor importance considering the estimation of CL_out_ to be 425 µL/min/g brain. The inter-individual variation was high with 134%, which was probably due to its low permeability into brain and the resultant low precision. Avdeef et al. measured atenolol K_in_ (unidirectional transfer constant into brain) under different pH values and concentrations, using the technique of in situ rat brain perfusion [34]. The K_in_, which is similar to CL_in_ for compounds with low permeability, was 1.8 µL/min/g brain for atenolol at 61.7 µM and pH 7.4, which is nearly 10% of the CL_in_ estimated from the model in the current study based on in vivo data. However, their study also showed high inter-individual variation in K_in_ with coefficient of variation (CV) ranging from 33.3% up to 1540% among the dosing groups. In another study published by Agon et al., positron emission tomography (PET) was used to monitor the brain uptake of atenolol in dogs after an i.v. bolus dose of 1.25 or 0.125 mg/kg [35]. By modeling the PET data, K_in_ was estimated ranging from 0.7 to 1.5 µL/min/g brain and the rate constant out of brain (k_out_) ranged from 0.0070 to 0.0151/min. Because of a lack of dog V_u,brain_ information, CL_out_ cannot be extracted from this PET study. However, it should be noted that instead of decreasing with blood concentration, the total drug concentration in dog brain remained at a stable level during the 90 min following the bolus dose. Given the low f_u,brain_ of atenolol due to its hydrophilic property, the difference in the profile of atenolol brain concentration between rats and dogs suggests a species difference in the BBB transport of atenolol.

By correcting for the surface area of endothelial cells in brain (100 cm^2^/g brain) [12], S-atenolol CL_in_ and Cl_out_ estimated from the model correspond to 2.83 × 10^−6^ and 70.8 × 10^−6^ cm/s of permeability coefficients into and out of rat brain, respectively. The permeability into brain was comparable to the P_app_ value (apparent in vitro transcellular permeability coefficient) assessed from an in vitro BBB model using primary rat brain endothelial cells, pericytes, and astrocytes (2.49 × 10^−6^ cm/s) [5]. Compared to the above model, the P_app_ values from in vitro BBB models composed of only brain microvessel endothelial cells were higher with 48.5 × 10^−6^ cm/s for bovine (BBMEC) and 9.78 × 10^−6^ cm/s for human (hBMEC) [36, 37]. The reported P_app_ values from other in vitro cell models bearing tight junctions for both A–B and B–A directions were in the range of 0.18 × 10^−6^ − 11 × 10^−6^ cm/s for Caco-2 cells and 0.13 × 10^−6^ − 0.8 × 10^−6^ cm/s for MDCKII (Madin-Darby canine kidney II cells) [37–40]. Although showing large inter-laboratory variation, these values and ranges are lower than the out-of-brain permeability estimated in the current study (70.8 × 10^−6^ cm/s), also suggesting the involvement of transporters in removing atenolol from the brain. Compared to the penetration permeability into the brain, atenolol exhibited higher intestinal absorption permeability based on in situ intestinal perfusion (5.5 × 10^−6^ cm/s for rats and 15 × 10^−6^ cm/s for human) [4, 41], which may be due to different characteristics of tight junctions and/or expression/function of related transporters.

Although being the most hydrophilic beta blocker, atenolol shows a much higher CL_in_ than sucrose (17.0 vs. 0.3 µL/min/g brain) [24]. Thus, the unbound profile of sucrose brain concentration was simulated to illustrate the unbound brain concentration–time profile of low intrinsic permeability (i.e. due to physicochemical property). As shown in Fig. 7c with CL_in_ and CL_out_ being the same and as low as 0.3 µL/min/g brain, the unbound brain concentration increases very slowly taking approximately 3.7 days to achieve 90% steady state. The ratio of C_u,brain_ to C_u,blood_ is only 25% at 12 h, indicating the very long time that would be needed to reach equal concentrations for a compound with such low intrinsic BBB permeability, (which therefore, in practice, is never measured at true equilibrium time points) and also showing a slower decline in unbound brain concentrations relative to unbound blood concentrations. Unlike the results of sucrose with low intrinsic permeability, the simulation of atenolol in Fig. 7b showed lower unbound concentration in brain than in blood at steady state, indicating the involvement of efflux transporter(s) in decreasing atenolol’s K_p,uu,brain_ value. In summary, the atenolol delivery to the brain is limited by the extent but not the rate of BBB transport.

In addition to K_p,uu,brain_ that is related to drug transport at the BBB, f_u,brain_ and V_u,brain_ are important measures to understand drug distribution within the brain, describing the intra-brain distribution [12]. Drug f_u,brain_ describes nonspecific binding within brain tissue while V_u,brain_ also describes intracellular distribution due to other reasons like transporters at some brain cell membranes. Similar to the nonspecific protein binding in plasma, hydrophilic drugs generally have low binding in brain homogenate [42]. From the equilibrium dialysis, atenolol had an f_u,brain_ of 0.875 ± 0.067. In contrast, propranolol has extensive nonspecific binding in brain homogenate with an f_u,brain_ of 0.029 [23]. If drug is evenly distributed within the brain parenchymal fluid, V_u,brain_ is close to the water volume of brain (0.8 mL/g brain). If drug is mainly is distributed inside brain cells or bound to brain tissues, V_u,brain_ tends to be larger than 0.8 mL/g brain [12]. The V_u,brain_ of S-atenolol estimated from microdialysis and whole brain measurements was 0.686 ± 0.104 mL/g brain, indicating no effects of transporters at the brain cells on the drug intra-brain distribution, or that there are transporters with counteractive functions transporting the drug in both the inward and outward directions at the same clearances across brain cell membrane. The latter is however much less likely.

It should be noted that it is the unbound, free drug rather than the bound drug that directly interacts with pharmacological targets. As a result, unbound drug concentration is more relevant to drug therapeutic effect instead of total drug in brain. In addition, the unbound drug concentration in brain ECF rather than total concentration of drug in brain tissue is more relevant in understanding drug transport across BBB because the total concentration of drug is confounded by ECF-ICF and/or nonspecific binding equilibration (as characterized by V_u,brain_ and f_u,brain_). The conclusion about BBB transport based on total drug concentrations in brain could therefore be misleading [43]. Thus, the K_p,uu,brain_ based on unbound concentration in plasma and brain ECF at steady state is a more clinically relevant measure to quantify drug transport at the BBB than rate measurements.

Our results suggest that some transporters actively eliminate atenolol from the brain, however no reports have been found to relate any possible BBB transporters with atenolol efflux. However, it was reported that fruit juices reduced the intestinal absorption of atenolol. The C_max_ and AUC were decreased by 49% and 40%, respectively, by orange juice, and 68% and 81%, respectively, by apple juice, based on pharmacokinetic studies in human subjects [44, 45]. There is some controversy in the literature about the transporters responsible for the interaction between atenolol and fruit juices. The organic anion transporting polypeptide 1A2 (OATP1A2) is suggested to be responsible of the atenolol uptake in the OATP1A2-expressed *X. laevis* oocytes [46]. However, another study by Mimura et al. suggested that organic cation transporter 1 (OCT1) rather than OATP probably contributes to the interaction between atenolol and flavonoids in fruit juices [47]. It was also reported that hOCT2 at the basolateral membrane of kidney tubules lead to renal active secretion of atenolol [48]. Furthermore, the study performed by Yin et al. suggested that atenolol is also a substrate of multidrug and toxin extrusion proteins (hMATE-1 and hMATE2-K) located at the apical membrane of renal tubule, thus contributing to the elimination of atenolol from blood to urine together with OCT2 [49]. Among these possible transporters for atenolol, only OATP has been found expressed at the BBB with bidirectional transport [50, 51]. OCT2 was also found to be expressed at the apical membrane of the blood-choroid plexus interface (i.e., CSF-facing), which may be relevant for efflux transport of substrates from cerebrospinal fluid to blood [52].

In addition to the solute carrier family (SLC), several members belonging to the ATP-binding cassette (ABC) transporter family are well known efflux transporters at the BBB with a wide range of substances, including P-glycoprotein (Pgp), multidrug resistance protein (MRP), and breast cancer resistance protein (BCRP) [53]. Studies are limited in evaluating the potential of atenolol as a substrate of MRP and BCRP, while controversial results have been reported for the role of brain and intestinal Pgp on atenolol efflux. Kallem et al. reported that coadministration of elacridar, a Pgp inhibitor, did not significantly change the total brain to plasma concentration ratio (K_p,brain_) or brain-to-plasma AUC ratio of atenolol in rats and mice [54]. An in situ intestinal perfusion study showed that verapamil, a Pgp inhibitor, did not change the absorption or intestinal permeability of atenolol [55, 56]. Similar conclusions that atenolol is not a Pgp substrate were drawn from in vitro studies using Caco-2 or Pgp transfected cell lines [40, 57]. On the other hand, Pgp inhibitors (cyclosporin and itraconazole) were reported to slightly increase the absorption rate and bioavailability of atenolol [58, 59]. However, these inhibitors are not specific and also act on other transporters. In addition, polarized transport of atenolol was found in a Pgp-transfected IPEC-J2 cell lines and Caco-2 cell with an efflux ratio of 3.5 and 2.3, respectively, which were decreased by addition of Pgp inhibitors (zosuquidar and verapamil) [60, 61]. In a collaborative study comparing Caco-2 cells from 10 laboratories, atenolol showed highly variable permeability and its efflux ratios ranged from 0.18 to 3.76, indicating the possibility of an involvement of transporter-mediated transport [38]. In summary, it is not clear which transporter(s) are responsible for the efflux of atenolol from brain, even though more solid evidence of transporter involvement have been found related to the intestinal absorption and renal secretion of atenolol.

## Conclusions

The present study systematically evaluated the extent of S-atenolol distribution into and within the brain using microdialysis, and strongly suggests an involvement of carrier-mediated efflux of S-atenolol at the BBB, in addition to passive diffusion. Although it is currently unclear which transporter (or transporters) is responsible for atenolol efflux transport at the BBB, it is likely not appropriate to use atenolol as a model drug for paracellular transport or passive diffusion. For any other candidate as a model drug of passive diffusion at the BBB, measurement of K_p,uu,brain_ based on unbound concentrations at steady state is useful to detect potential involvement of transporters in the BBB transport. The likely transporters may have different expression levels and functions in other organs (e.g. intestine and kidney), thus the importance of carrier-mediated transport is likely different depending on the organ studied.

## Abbreviations

AUC_u_: unbound drug concentration–time profiles
BBB: blood-brain barrier
BCRP: breast cancer resistance protein
C_in,ATD7_: concentrations of atenolol-D7 in perfusate
C_out,ATD7_: concentrations of atenolol-D7 in dialysate
C_u,brain_: unbound drug concentration in brain ECF
C_u,blood_: unbound drug concentrations in blood
C_u,ss,brainECF_: unbound drug concentrations in brain ECF at steady state
C_u,ss,plasma_: unbound drug concentrations in plasma at steady state
CL_in_: influx clearance into brain
CL_out_: efflux clearance from brain
CV: coefficient of variation
ECF: extracellular fluid
f_u,brain_: unbound drug fraction in brain homogenate
f_u,hD_: unbound drug fraction in diluted brain homogenate
f_u,p_: unbound drug fraction in plasma
ICF: intracellular fluid
K_in_: unidirectional transfer constant into brain
K_p,uu,brain_: unbound equilibrium partition coefficient
LC–MS/MS: liquid chromatography coupled with tandem mass spectrometry
hMATE: multidrug and toxin extrusion proteins
MRP: multidrug resistance protein
OATP: organic anion transporting polypeptide
OCT: organic cation transporter
PAES: polyarylethersulphone
Pgp: P-glycoprotein
r^2^: coefficients of determination
R_bl-p_: blood-to-plasma concentration ratio
RSE: relative standard error
t_1/2,brain_: half-lives
V_u,brain_: unbound volume of distribution in brain
V_bl_: volume of vascular space in brain

## References

[1] Ong HT (2007). Beta blockers in hypertension and cardiovascular disease. BMJ.

[2] Pearson AA, Gaffney TE, Walle T, Privitera PJ (1989). A stereoselective central hypotensive action of atenolol. J Pharmacol Exp Ther.

[3] Mehvar R, Brocks DR (2001). Stereospecific pharmacokinetics and pharmacodynamics of beta-adrenergic blockers in humans. J Pharm Pharm Sci.

[4] Lennernas H (1998). Human intestinal permeability. J Pharm Sci.

[5] Nakagawa S, Deli MA, Kawaguchi H, Shimizudani T, Shimono T, Kittel A, Tanaka K, Niwa M (2009). A new blood-brain barrier model using primary rat brain endothelial cells, pericytes and astrocytes. Neurochem Int.

[6] Smith D, Artursson P, Avdeef A, Di L, Ecker GF, Faller B, Houston JB, Kansy M, Kerns EH, Kramer SD, Lennernas H, van de Waterbeemd H, Sugano K, Testa B (2014). Passive lipoidal diffusion and carrier-mediated cell uptake are both important mechanisms of membrane permeation in drug disposition. Mol Pharm.

[7] Abbott NJ, Ronnback L, Hansson E (2006). Astrocyte-endothelial interactions at the blood-brain barrier. Nat Rev Neurosci.

[8] Bickel U (2005). How to measure drug transport across the blood–brain barrier. NeuroRx.

[9] Oldendorf WH (1970). Measurement of brain uptake of radiolabeled substances using a tritiated water internal standard. Brain Res.

[10] Takasato Y, Rapoport SI, Smith QR (1984). An in situ brain perfusion technique to study cerebrovascular transport in the rat. Am J Physiol.

[11] Hammarlund-Udenaes M (2014). In vivo approaches to assessing the blood–brain barrier. Top Med Chem Ser.

[12] Hammarlund-Udenaes M, Friden M, Syvanen S, Gupta A (2008). On the rate and extent of drug delivery to the brain. Pharm Res.

[13] Lindqvist A, Jonsson S, Hammarlund-Udenaes M (2016). Exploring factors causing low brain penetration of the opioid peptide DAMGO through experimental methods and modeling. Mol Pharm.

[14] Delange ECM, Danhof M, Deboer AG, Breimer DD (1994). Critical factors of intracerebral microdialysis as a technique to determined the pharmacokinetics of drugs in rat-brain. Brain Res.

[15] Hammarlund-Udenaes M, Paalzow LK, de Lange EC (1997). Drug equilibration across the blood–brain barrier–pharmacokinetic considerations based on the microdialysis method. Pharm Res.

[16] Sadiq MW, Borgs A, Okura T, Shimomura K, Kato S, Deguchi Y, Jansson B, Bjorkman S, Terasaki T, Hammarlund-Udenaes M (2011). Diphenhydramine active uptake at the blood–brain barrier and its interaction with oxycodone in vitro and in vivo. J Pharm Sci.

[17] Bouw MR, Hammarlund-Udenaes M (1998). Methodological aspects of the use of a calibrator in in vivo microdialysis-further development of the retrodialysis method. Pharm Res.

[18] Bengtsson J, Bostrom E, Hammarlund-Udenaes M (2008). The use of a deuterated calibrator for in vivo recovery estimations in microdialysis studies. J Pharm Sci.

[19] Bostrom E, Simonsson US, Hammarlund-Udenaes M (2006). In vivo blood–brain barrier transport of oxycodone in the rat: indications for active influx and implications for pharmacokinetics/pharmacodynamics. Drug Metab Dispos.

[20] Sadiq MW, Bostrom E, Keizer R, Bjorkman S, Hammarlund-Udenaes M (2013). Oxymorphone active uptake at the blood–brain barrier and population modeling of its pharmacokinetic-pharmacodynamic relationship. J Pharm Sci.

[21] Bickel U, Schumacher OP, Kang YS, Voigt K (1996). Poor permeability of morphine 3-glucuronide and morphine 6-glucuronide through the blood–brain barrier in the rat. J Pharmacol Exp Ther.

[22] Taylor EA, Turner P (1981). The distribution of propranolol, pindolol and atenolol between human-erythrocytes and plasma. Brit J Clin Pharmaco.

[23] Friden M, Bergstrom F, Wan H, Rehngren M, Ahlin G, Hammarlund-Udenaes M, Bredberg U (2011). Measurement of unbound drug exposure in brain: modeling of pH partitioning explains diverging results between the brain slice and brain homogenate methods. Drug Metab Dispos.

[24] Ennis SR, Betz AL (1986). Sucrose permeability of the blood-retinal and blood-brain barriers. Effects of diabetes, hypertonicity, and iodate. Invest Ophthalmol Vis Sci.

[25] Neildwyer G, Bartlett J, Mcainsh J, Cruickshank JM (1981). Beta-adrenoceptor blockers and the blood–brain-barrier. Brit J Clin Pharmaco.

[26] Sun D, Lennernas H, Welage LS, Barnett JL, Landowski CP, Foster D, Fleisher D, Lee KD, Amidon GL (2002). Comparison of human duodenum and Caco-2 gene expression profiles for 12,000 gene sequences tags and correlation with permeability of 26 drugs. Pharm Res.

[27] Camenisch G, Alsenz J, van de Waterbeemd H, Folkers G (1998). Estimation of permeability by passive diffusion through Caco-2 cell monolayers using the drugs’ lipophilicity and molecular weight. Eur J Pharm Sci.

[28] Artursson P (1990). Epithelial transport of drugs in cell-culture. 1. A model for studying the passive diffusion of drugs over intestinal absorptive (Caco-2) cells. J Pharm Sci.

[29] Cheng Z, Zhang J, Liu H, Li Y, Zhao Y, Yang E (2010). Central nervous system penetration for small molecule therapeutic agents does not increase in multiple sclerosis- and Alzheimer’s disease-related animal models despite reported blood–brain barrier disruption. Drug Metab Dispos.

[30] Scherrmann JM (2002). Drug delivery to brain via the blood–brain barrier. Vascul Pharmacol.

[31] Giacomini KM, Huang SM, Tweedie DJ, Benet LZ, Brouwer KL, Chu X, Dahlin A, Evers R, Fischer V, Hillgren KM, Hoffmaster KA, Ishikawa T, Keppler D, Kim RB, Lee CA, Niemi M, Polli JW, Sugiyama Y, Swaan PW, Ware JA, Wright SH, Yee SW, Zamek-Gliszczynski MJ, Zhang L, International Transporter C (2010). Membrane transporters in drug development. Nat Rev Drug Discov.

[32] Rosenberg GA, Kyner WT, Estrada E (1980). Bulk flow of brain interstitial fluid under normal and hyperosmolar conditions. Am J Physiol.

[33] Cserr HF, Cooper DN, Milhorat TH (1977). Flow of cerebral interstitial fluid as indicated by the removal of extracellular markers from rat caudate nucleus. Exp Eye Res.

[34] Avdeef A, Sun N (2011). A new in situ brain perfusion flow correction method for lipophilic drugs based on the pH-dependent Crone-Renkin equation. Pharm Res.

[35] Agon P, Goethals P, Van Haver D, Kaufman JM (1991). Permeability of the blood–brain barrier for atenolol studied by positron emission tomography. J Pharm Pharmacol.

[36] Eigenmann DE, Jahne EA, Smiesko M, Hamburger M, Oufir M (2016). Validation of an immortalized human (hBMEC) in vitro blood–brain barrier model. Anal Bioanal Chem.

[37] Hakkarainen JJ, Jalkanen AJ, Kaariainen TM, Keski-Rahkonen P, Venalainen T, Hokkanen J, Monkkonen J, Suhonen M, Forsberg MM (2010). Comparison of in vitro cell models in predicting in vivo brain entry of drugs. Int J Pharm.

[38] Hayeshi R, Hilgendorf C, Artursson P, Augustijns P, Brodin B, Dehertogh P, Fisher K, Fossati L, Hovenkamp E, Korjamo T, Masungi C, Maubon N, Mols R, Mullertz A, Monkkonen J, O’Driscoll C, Oppers-Tiemissen HM, Ragnarsson EG, Rooseboom M, Ungell AL (2008). Comparison of drug transporter gene expression and functionality in Caco-2 cells from 10 different laboratories. Eur J Pharm Sci.

[39] Wang Q, Rager JD, Weinstein K, Kardos PS, Dobson GL, Li JB, Hidalgo IJ (2005). Evaluation of the MDR-MDCK cell line as a permeability screen for the blood-brain barrier. Int J Pharm.

[40] Gartzke D, Delzer J, Laplanche L, Uchida Y, Hoshi Y, Tachikawa M, Terasaki T, Sydor J, Fricker G (2015). Genomic knockout of endogenous canine P-glycoprotein in wild-type, human P-glycoprotein and human BCRP transfected MDCKII cell lines by zinc finger nucleases. Pharm Res.

[41] Fagerholm U, Johansson M, Lennernas H (1996). Comparison between permeability coefficients in rat and human jejunum. Pharm Res.

[42] Wan H, Rehngren M, Giordanetto F, Bergstrom F, Tunek A (2007). High-throughput screening of drug-brain tissue binding and in silico prediction for assessment of central nervous system drug delivery. J Med Chem.

[43] Chen X, Keep RF, Liang Y, Zhu HJ, Hammarlund-Udenaes M, Hu Y, Smith DE (2017). Influence of peptide transporter 2 (PEPT2) on the distribution of cefadroxil in mouse brain: a microdialysis study. Biochem Pharmacol.

[44] Lilja JJ, Raaska K, Neuvonen PJ (2005). Effects of orange juice on the pharmacokinetics of atenolol. Eur J Clin Pharmacol.

[45] Jeon H, Jang IJ, Lee S, Ohashi K, Kotegawa T, Ieiri I, Cho JY, Yoon SH, Shin SG, Yu KS, Lim KS (2013). Apple juice greatly reduces systemic exposure to atenolol. Br J Clin Pharmacol.

[46] Kato Y, Miyazaki T, Kano T, Sugiura T, Kubo Y, Tsuji A (2009). Involvement of influx and efflux transport systems in gastrointestinal absorption of celiprolol. J Pharm Sci.

[47] Mimura Y, Yasujima T, Ohta K, Inoue K, Yuasa H (2015). Atenolol transport by organic cation transporter 1 and its interference by flavonoids. Drug Metab Rev.

[48] Ciarimboli G, Schroter R, Neugebauer U, Vollenbroker B, Gabriels G, Brzica H, Sabolic I, Pietig G, Pavenstadt H, Schlatter E, Edemir B (2013). Kidney transplantation down-regulates expression of organic cation transporters, which translocate beta-blockers and fluoroquinolones. Mol Pharm.

[49] Yin J, Duan HC, Shirasaka Y, Prasad B, Wang J (2015). Atenolol renal secretion is mediated by human organic cation transporter 2 and multidrug and toxin extrusion proteins. Drug Metab Dispos.

[50] Ronaldson PT, Davis TP (2013). Targeted drug delivery to treat pain and cerebral hypoxia. Pharmacol Rev.

[51] Westholm DE, Rumbley JN, Salo DR, Rich TP, Anderson GW (2008). Organic anion-transporting polypeptides at the blood-brain and blood-cerebrospinal fluid barriers. Curr Top Dev Biol.

[52] Sweet DH, Miller DS, Pritchard JB (2001). Ventricular choline transport: a role for organic cation transporter 2 expressed in choroid plexus. J Biol Chem.

[53] Loscher W, Potschka H (2005). Role of drug efflux transporters in the brain for drug disposition and treatment of brain diseases. Prog Neurobiol.

[54] Kallem R, Kulkarni CP, Patel D, Thakur M, Sinz M, Singh SP, Mahammad SS, Mandlekar S (2012). A simplified protocol employing elacridar in rodents: a screening model in drug discovery to assess P-gp mediated efflux at the blood brain barrier. Drug Metab Lett.

[55] Mols R, Brouwers J, Schinkel AH, Annaert P, Augustijns P (2009). Intestinal perfusion with mesenteric blood sampling in wild-type and knockout mice: evaluation of a novel tool in biopharmaceutical drug profiling. Drug Metab Dispos.

[56] Brouwers J, Mols R, Annaert P, Augustijns P (2010). Validation of a differential in situ perfusion method with mesenteric blood sampling in rats for intestinal drug interaction profiling. Biopharm Drug Dispos.

[57] Doppenschmitt S, Spahn-Langguth H, Regardh CG, Langguth P (1999). Role of P-glycoprotein-mediated secretion in absorptive drug permeability: an approach using passive membrane permeability and affinity to P-glycoprotein. J Pharm Sci.

[58] Terao T, Hisanaga E, Sai Y, Tamai I, Tsuji A (1996). Active secretion of drugs from the small intestinal epithelium in rats by P-glycoprotein functioning as an absorption barrier. J Pharm Pharmacol.

[59] Lilja JJ, Backman JT, Neuvonen PJ (2005). Effect of itraconazole on the pharmacokinetics of atenolol. Basic Clin Pharmacol Toxicol.

[60] Saaby L, Helms HC, Brodin B (2016). IPEC-J2 MDR1, a novel high-resistance cell line with functional expression of human P-glycoprotein (ABCB1) for drug screening studies. Mol Pharm.

[61] Augustijns P, Mols R (2004). HPLC with programmed wavelength fluorescence detection for the simultaneous determination of marker compounds of integrity and P-gp functionality in the Caco-2 intestinal absorption model. J Pharm Biomed Anal.

